# Supplementary material for: The Construction and Exploration of a Comprehensive MicroRNA Centered Regulatory Network in Foxtail Millet (Setaria italica L.)
Source: Front Plant Sci. 2022 May 6;13:848474. doi: 10.3389/fpls.2022.848474 (PMC9121102; doi:10.3389/fpls.2022.848474)
Supplement: Supplementary file 4 [file Data_Sheet_4.zip › Sit-miR394_Seita.9G502900.1_679_TPlot.pdf]

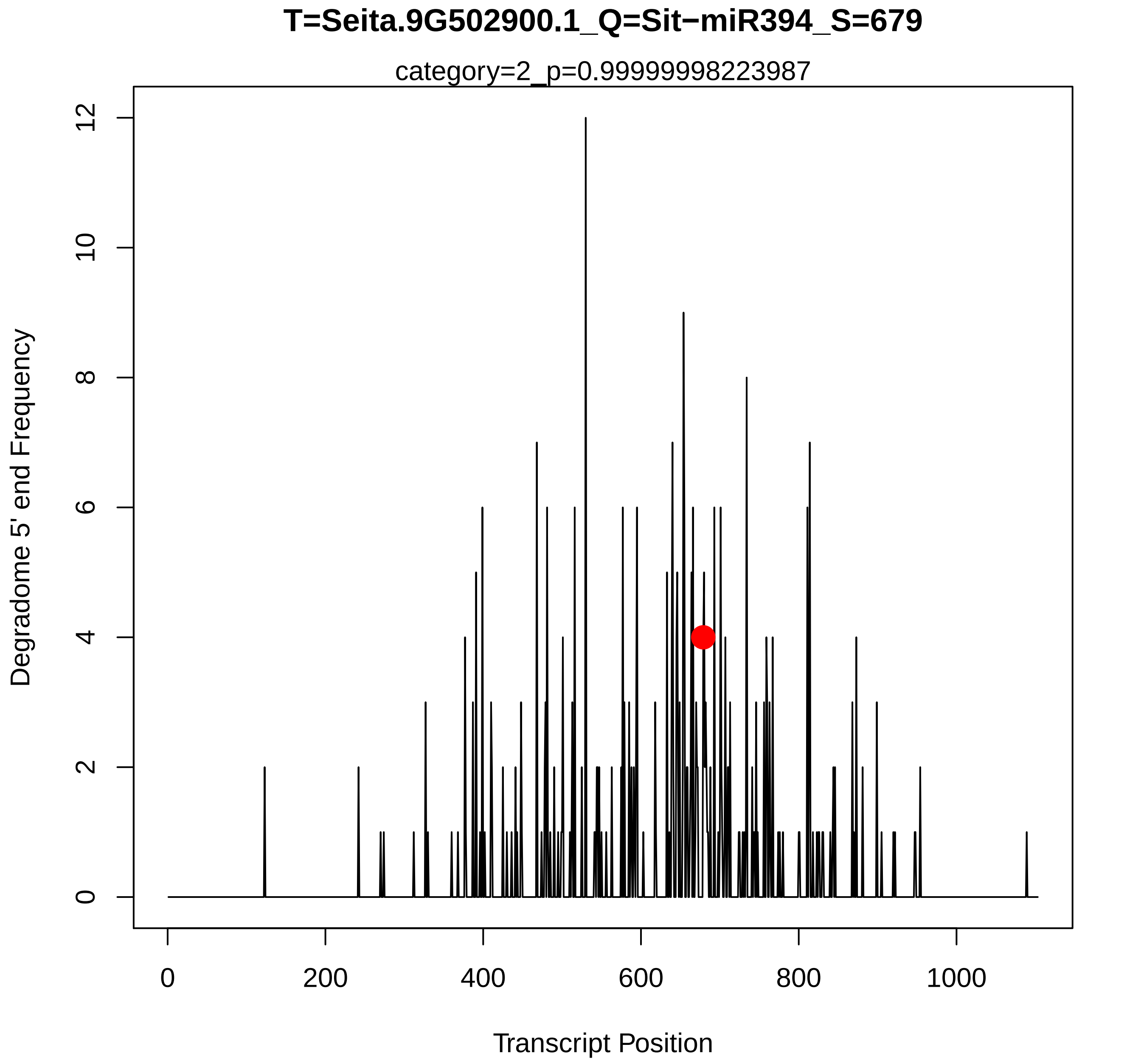
T=Seita.9G502900.1_Q=Sit−miR394_S=679
category=2_p=0.99999998223987
Degradome 5' end Frequency
0
2
4
6
8
10
12
0
200
400
600
800
1000
Transcript Position
